# Requirement for PINCH in skeletal myoblast differentiation

**DOI:** 10.1007/s00441-022-03701-1

**Published:** 2022-11-17

**Authors:** Huimin Liao, Fei Wang, Ke Lu, Xiaolei Ma, Jie Yan, Lina Luo, Yunfu Sun, Xingqun Liang

**Affiliations:** Key Laboratory of Arrhythmia, Ministry of Education, East Hospital, Tongji University School of Medicine, 150 Jimo Road, Shanghai, 200120 China

**Keywords:** PINCH, Integrin pathway, Skeletal myogenesis, Cytoskeleton organization, Cell fusion

## Abstract

PINCH, an adaptor of focal adhesion complex, plays essential roles in multiple cellular processes and organogenesis. Here, we ablated PINCH1 or both of PINCH1 and PINCH2 in skeletal muscle progenitors using MyoD-Cre. Double ablation of PINCH1 and PINCH2 resulted in early postnatal lethality with reduced size of skeletal muscles and detachment of diaphragm muscles from the body wall. PINCH mutant myofibers failed to undergo multinucleation and exhibited disrupted sarcomere structures. The mutant myoblasts in culture were able to adhere to newly formed myotubes but impeded in cell fusion and subsequent sarcomere genesis and cytoskeleton organization. Consistent with this, expression of integrin β1 and some cytoskeleton proteins and phosphorylation of ERK and AKT were significantly reduced in PINCH mutants. However, N-cadherin was correctly expressed at cell adhesion sites in PINCH mutant cells, suggesting that PINCH may play a direct role in myoblast fusion. Expression of MRF4, the most highly expressed myogenic factor at late stages of myogenesis, was abolished in PINCH mutants that could contribute to observed phenotypes. In addition, mice with PINCH1 being ablated in myogenic progenitors exhibited only mild centronuclear myopathic changes, suggesting a compensatory role of PINCH2 in myogenic differentiation. Our results revealed a critical role of PINCH proteins in myogenic differentiation.

## Introduction

Skeletal muscle is composed of bundles of myofibers sheathed by extracellular matrix networks and is responsible for force generation, contraction, and body movement. Malformation of skeletal muscle during embryonic development results in congenital myopathies, a heterogeneous group of primary muscle disorders characterized by muscle weakness and a variety of myopathic changes in muscle biopsies including nemaline rods, central core, multi-mini core, and centralized nuclei (Jungbluth et al. 2018; Mercuri and Muntoni 2012, 2013; Vasli et al. 2017). Disease mechanisms of congenital myopathies remain unclear. Therefore, elucidation of the molecular mechanisms underlying skeletal myogenesis will offer new insights into the pathogenesis of myopathies and is critical to the development of novel therapies.

Skeletal myogenesis is a multistep process regulated by distinct signaling pathways (e.g., Wnt, FGF, and Notch pathways) and a network of myogenic transcription factors (Bober et al. 1991; Braun et al. 1994; Bryson-Richardson and Currie 2008; Kassar-Duchossoy et al. 2004). Pax3 and Pax7, via their downstream myogenic regulatory factors (MRF5, MyoD), are required for the specification and determination of myogenic progenitors and myoblasts (Daston et al. 1996; Seale et al. 2000). Myoblasts initiate the expression of other two myogenic factors MRF4 and MyoG1 that promote muscle differentiation by upregulation of genes encoding muscle-specific contractile and structural proteins. Mononucleated myoblasts eventually exit the cell cycle and fuse to form multinucleated myofibers (Du 1997; Tajbakhsh et al. 1998). The mature myofibers are filled with well-aligned myofibrils made up of sarcomeres, which in turn are interconnected with the cell membrane (sarcolemma), nucleus, and organelles via actin filament networks. One of the features of mature myofibers is the peripheral positioning of the nucleus underneath the cell membrane. Nuclear shape and position of myofibers are controlled by coordinated action of both actin and microtubule-based cytoskeleton systems and by the interaction of cytoskeleton and nucleoskeleton. This unique cytoskeletal architecture of muscle cells is essential for mechanical sensing and transduction, gene expression, and contractile function. However, molecular mechanisms regulating cytoskeletal assembly and integrity of skeletal muscle are not fully understood.

Integrin signaling pathways mediate cell–matrix interactions and play essential roles in multiple cellular processes during embryonic development. Integrin-focal adhesion complex mediates the crosstalk between extracellular matrix (ECM) and intracellular signaling pathways and cytoskeleton, and is essential for ECM deposition and cytoskeleton organization and remodeling, and functions as a key mechanical sensor and anchorage sites for cell adhesion and migration. Integrin pathways play an important role in muscle development and function, and disruptions of integrin-mediated adhesion in skeletal muscles result in myopathies in humans and mice (Sparrow and Schock 2009). Receptors of the β1 integrin family are required for the fusion of mononucleated myoblasts and subsequent assembly of cytoskeleton (Schwander et al. 2003). Blocking integrin α6β1 binding to laminin inhibits myotome formation, and mutations in the Integrin α7 gene cause congenital myopathy in both patients and mice (Bajanca 2006; Hirabayashi et al. 1998; Rooney et al. 2006). Muscle-specific ablation of ILK or Talin1 similarly causes progressive muscular dystrophy with a detachment of myotendinous junctions (Conti et al. 2008; Wang et al. 2008).

PINCH is a key component of focal adhesion in complex with ILK and Parvin, and plays a critical role in mediating Integrin signaling (Braun et al. 2003; Xu et al. 2016). Genetic studies have revealed essential roles of PINCH during embryonic development and in morphogenesis of multiple tissues and organs, similar with that of ILK and β1-Integrin (Legate et al. 2006; Liang et al. 2009a, b, 2005). In *C. elegans*, loss of UNC-97 (PINCH) results in impaired muscular adhesion junctions and body wall muscle deformity (Hobert et al. 1999; Norman et al. 2007). Similarly, mutation of PINCH in muscle cells of drosophila leads to muscle detachment (Clark et al. 2003). Myocardial-specific ablation of PINCH1 in mice causes dilated cardiomyopathy with disrupted sarcomere structures, aberrant expression of cytoskeleton-associated proteins, and reduced Akt phosphorylation and activation (Liang et al. 2009b). So far, the role of PINCH in skeletal myogenesis in mammals remains unclear.

In this study, we ablated PINCH1 and PINCH2 specifically in skeletal muscle progenitor cells using MyoD-Cre mice. PINCH1 and PINCH2 double mutant mice (PINCH mutants) exhibited congenital skeletal myopathy of variable severity. Ablation of PINCH1 resulted in postnatal growth retardation and hypoplastic and centrally nucleated myofibers. Although, PINCH2-deficient mice are viable and fertile and display no phenotype (Stanchi et al. 2005). Double ablation of PINCH1 and PINCH2 caused early postnatal lethality and defects in myoblast fusion and subsequent myofibril assembly. Western blot and immunostaining revealed reduced phosphorylation and activation of AKT and ERK1/2 and a significant reduction in the expression of proteins associated with muscle cytoskeleton and contraction. We found that MRF4, the most highly expressed myogenic factor at late stages of myogenesis, was abolished in PINCH mutant muscles that could contribute to impaired terminal differentiation of muscle fibers.

## Materials and methods

### Animals

To generate skeletal muscle-specific PINCH1 knock-out mice (MyoD-Cre, PINCH1^f/f^, PINCH2^+/+^, SMUT) with C57BL/6 J background, we crossed MyoD-Cre, PINCH1^f/+^, PINCH2^+/+^ males with PINCH1^f/f^, and PINCH2^+/+^ females on Rosa26-LacZ background in order to trace Cre lineage. Muscle-specific PINCH1 and PINCH2 double knock-out mice (MyoD-Cre, PINCH^f/f^, PINCH2^−/−^, DMUT) were produced by crossing Myo-Cre, PINCH1^f/+^, PINCH2^−/−^ males with PINCH1^f/f^, and PINCH2^−/−^ females. Mice were housed in the Laboratory Animal Facility at Tongji University. All animal experiments were performed according to the guidelines for the Care and Use of Laboratory Animals (Ministry of Health, China, 1998) and monitored by the Institutional Animal Care and Use Committee of Tongji University School of Medicine.

### Histologic analysis

Mice were sacrificed and muscle tissues were dissected and fixed overnight in 4% paraformaldehyde at 4 °C, and paraffin-embedded. Sections were cut at 8–10 μm from paraffin blocks and stained with hematoxylin and eosin.

### Transmission electron microscopy (TEM)

The quadriceps muscles were processed for electron microscopy analysis as described (Liang et al. 2009b). Briefly, the quadriceps muscles of postnatal day 1 (P1) DMUT and control mice were fixed in 2.5% glutaraldehyde in PBS overnight at 4 ℃ and then immersed in 1% osmium tetroxide for 1 h at room temperature. After fixation, the samples were dehydrated in graduated alcohols and embedded with epoxy resin. Ultrathin sections longitudinally to the myofibrils were performed and stained with toluidine blue. Then, the sections were contrasted with uranyl acetate and lead citrate and subsequently examined in the transmission electron microscope (JEM-1230).

### Western blotting

For protein extraction, mice in P1 were sacrificed and dissected. Protein was extracted and prepared using the Protein Extraction kit (Keygen KGP250). The following antibodies were used: β1 integrins (1:500, Sigma-Aldrich, SAB4300655), ILK (1:1000, Sigma-Aldrich, I0783), Talin (1:100, Abcam, ab71333), N-cadherin (1:1000, Sigma-Aldrich, SAB5700640), Vinculin (1:2000, Sigma-Aldrich, V4139), Akt (1:200, Cell Signaling, #9267), Phospho-Akt (1:500, Cell Signaling, #9271), Erk1/2 (1:250, Cell Signaling, #4695), P-Erk1/2 (1:800, Cell Signaling, #4370), MRF4 (1:200, Invitrogen, PA5-120,339), Myosin (1: 200; Abcam, ab264490), Titin (1:250, Novus Biologicals, NB600-1206), Desmin (1: 80; Abcam, ab32362). Western Blot was performed as described. Relative expression levels of proteins were quantified by ImageJ 1.8.0 software.

### Isolation and culture of mouse primary myoblasts

The mouse primary myoblasts were isolated as previously described with slight modifications (Miller and Hollenbach 2007). Primary myoblasts were isolated from the hindlimb muscle of E18.5 embryos and digested with collagenase/dispase (1 mg/ml, Roche Applied Science, **#** 10,269,638,001) at 37 °C for 45 min. The cell suspension was passed through an 80 μm nylon mesh cell strainer, and cells were pre-plated in an uncoated 60 cm Corning culture dish for up to 1 h two times to remove fibroblasts. Untouched myoblasts were collected and cultured on collagen-coated coverslips (Fisherbrand, #12–545-80P) in proliferation medium (F-10 nutrient media (GIBCO, #12,390,035) with 20% fetal bovine serum (GIBCO, #10,099,141), 2.5 ng/ml basic fibroblast growth factor (GIBCO, PMG0031)) at 37 °C in 5% CO_2_. After 2 days, cultures were changed to a differentiation medium (DMEM (GIBCO, # 12,430,047) with 2% horse serum (GIBCO, #26,050,070)) for 3 days.

### Immunofluorescence

For immunostaining, samples were fixed in 4% PFA overnight, embedded in optimal cutting temperature compound (Leica, #4583) and cut at 10 μm per section with a Leica CM1860 microtome (Leica). Sections were incubated with primary antibodies overnight at 4 °C. The following primary antibodies were used: α-actinin (1:400, Sigma**-**Aldrich, A5044), N-cadherin (1:500, Sigma-Aldrich, SAB5700640). After washing in 0.25% TritonX-100 in PBS, sections were incubated with fluorescent-labeled antibodies (Molecular Probes, Invitrogen) for 2 h.

For cell staining, cultured myoblasts and myotubes were washed briefly with PBS and fixed in 4% paraformaldehyde for 20 min and then permeabilized with PBS containing 0.125% Triton X-100 for 30 min. After being washed 3 times with PBS, the cells were blocked with 5% BSA at room temperature for 30 min. The primary antibodies were applied at 4 °C overnight. The following primary antibodies were used: desmin (1: 50; Abcam, ab32362), Myosin (1: 100; Abcam, ab264490), Talin (1:50, Abcam, ab71333), and Vinculin (1:500, Sigma-Aldrich, V4139). After washing in 0.125% TritonX-100 in PBS, cells were incubated with fluorescent-labeled antibodies (Molecular Probes, Invitrogen) for 2 h and then visualized under a fluorescence microscope (Leica DM6000).

### Statistical analysis

Data are presented as mean ± SEM. Student *t*-test was used for 2-group comparisons. Differences were considered statistically significant at *p* < 0.05.

## Results

### Impaired myogenic differentiation and maturation in mice with PINCH1 being ablated in skeletal muscle progenitors

**Fig. 1:**
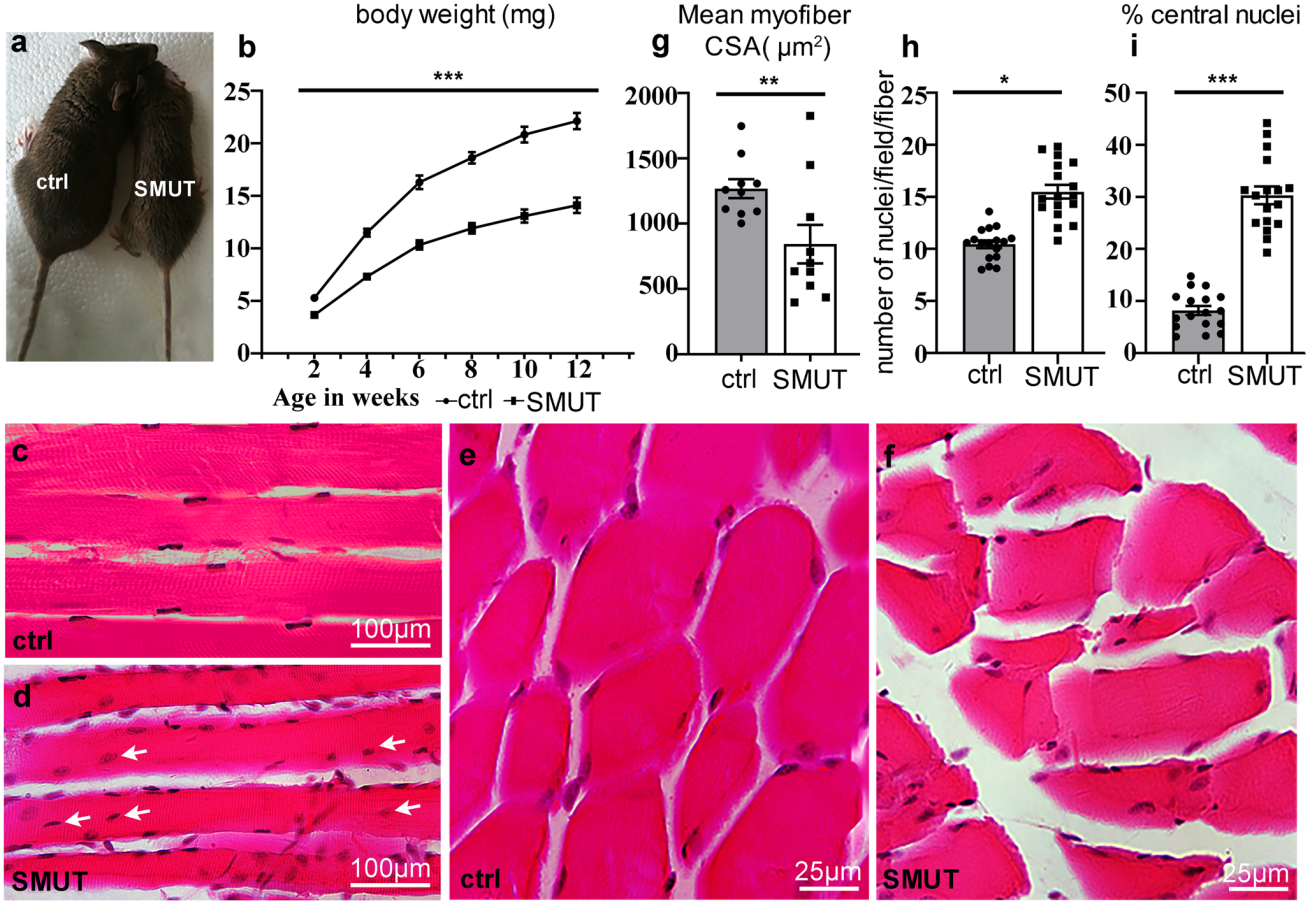
Single ablation of PINCH1 in skeletal muscle progenitors caused myopathic changes. **a**, **b** At different time points during postnatal growth, MyoD-Cre; PINCH1.^f/−^(SMUT) mice exhibited significant growth retardation with reduced height and body weight compared to control littermates (ctrl). (*n* = 6, *p* < 0.001, 2-tailed *t*-test). **c**–**g** H&E staining of sagittal **(c**, **d**) and transversal sections **(e**, **f**) of quadriceps femoris of SMUT and controls. **g** Analysis of mean cross-sectional area (CSA) of myofibers at the age of 12 weeks by measuring the CSA of all fibers in the entire section, using ImageJ 1.8.0 software; **h** Quantification of number of nuclei per myofiber per section area at postnatal 12 weeks; **i** Quantification of number of central nuclei expressed as percentage of total number of nuclei per fiber per section area at postnatal 12 weeks. (*n* = 16, error bars represent ± SEM, *p < 0.05, **p < 0.01, ***p < 0.001, 2-tailed *t*-test)

To investigate the specific role of PINCH in skeletal muscle development, we generated a mouse line in which PINCH1 was deleted in skeletal muscle progenitor cells using MyoD-Cre (Chen et al. 2005). PINCH1 mutant mice (MyoD-Cre, PINCH1^f/f^, SMUT) were born at expected mendelian ratio and appeared normal at birth. We measured the body weight of SMUT mice at different ages during postnatal growth, as compared with that of age-and sex-matched control littermates. During postnatal development, SMUT mice exhibited mild growth retardation with shorter body lengths and lower body weight compared to control littermates (Fig. 1a, b). PINCH1 mutants appeared less active, otherwise fertile, and lived a normal lifespan.

The skeletal muscles are composed of bundles of multinucleated myofibers, each of which is filled with myofibrils with its nuclei placed at the periphery underneath sarcolemma (Chal and Pourquie 2017; Roman and Gomes 2018). To investigate if there were any morphological change in SMUT skeletal muscles, we dissected the quadriceps muscles from 2-month adult SMUT mice and control littermates and performed H&E (Hematoxylin–Eosin staining) histological analyses. We found that ablation of PINCH1 in skeletal muscle progenitors resulted in a significant myopathic change. In longitudinal (Fig. 1c, d) and cross (Fig. 1f, g) sections, myofibers of control samples were compact and the nuclei were spindle-like and evenly located immediately below the plasma membrane (Fig. 1c, d). However, SMUT myofibers were loosely packed and significantly hypoplastic and variable in size. There was a significant decrease in average cross-sectional area (CSA) of SMUT myofibers compared to controls by measuring the CSA of all fibers in the entire section. (Fig. 1c–g). Nuclei of SMUT myofibers exhibited centrally localized, a feature of myopathies (Fig. 1d, arrow). The number of nuclei and percentage of centrally localized nuclei in SMUT myofibers were significantly increased compared to the control (Fig. 1h, i). These results suggested that deletion of PINCH1 in skeletal muscle progenitors leads to impaired terminal differentiation and maturation of multinucleated myofibers.

## Ablation of PINCH1 and PINCH2 resulted in reduced size of muscle fibers and impaired multinucleation

**Fig. 2:**
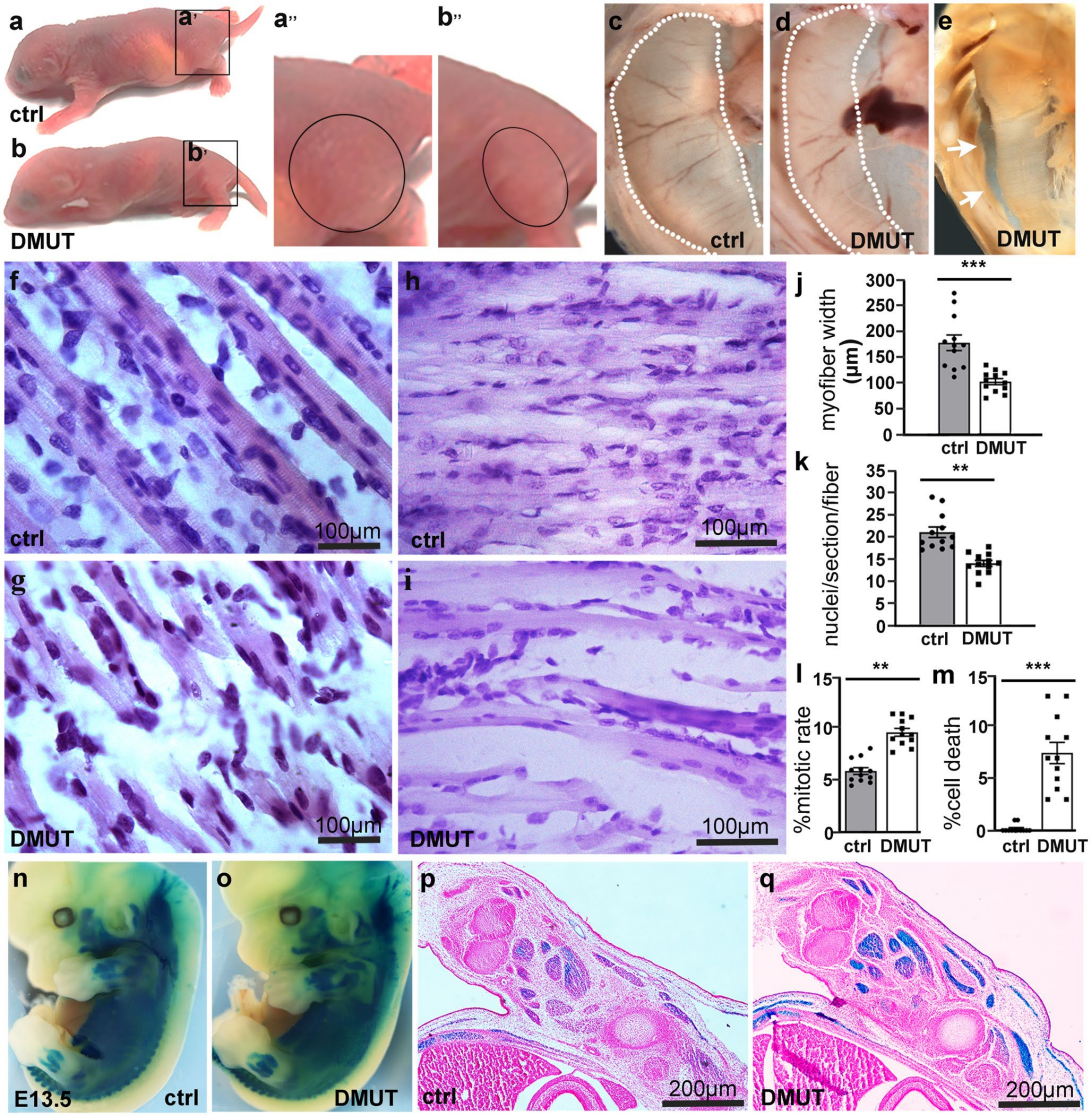
Ablation of PINCH1 and PINCH2 results in reduced size of muscle fibers and impaired multinucleation. **a**, **b** (a’’, b’’) A majority of PINCH1 and PINCH2 mutant mice (DMUT) died within 48 h, while all remaining mice would survive no more than 7–10 days. DMUT appeared to be slim with markedly reduced muscle masses compared to littermate control (ctrl). **c**–**e** Wholemount view of DMUT and control diaphragm muscles, showing marked hypotrophy **d **and detachment (**e**, arrow) of DMUT diaphragm muscles. **f**, **g** H&E-stained longitudinal sections of thigh **f**, **g **and diaphragm **h**, **i **muscles from postnatal day 1 (P1) DMUT mice and littermate controls. **j**–**m** Quantification of myofiber width **j**, number of nuclei **k**, mitotic nuclei **l **and cell death **m**. (*n* = 12, Error bars represent ± SEM, **p < 0.01, ***p < 0.001, 2-tailed *t*-test). **n**–**q** Wholemount and section β-gal staining of E13.5 DMUT and control mice staining showing overall shape and size of somites and muscle primordial groups were comparable between DMUT and control mice

Two PINCH isoforms (PINCH1 and 2) are expressed in mammals with partial functional redundancy (Liang et al. 2009a, b). Relative mild phenotype in PINCH1 mutants could be attributed in part to the compensatory role by PINCH2. To examine this possibility, we generated a muscle-specific PINCH1 and PINCH2 double knockout mouse line (MyoD-Cre, PINCH1^f/f^, PINCH2^−/−^, DMUT). A majority of DMUT mice would die within 48 h after birth, and all remaining mice die within 7–10 days (Fig. 2a, b). Neonatal DMUT pups appeared weak, slim with an empty stomach, suggesting defects in sucking and motor function. We found a marked reduction in the size of muscles in the back and thighs (Fig. 2a’, b’ boxed) in DMUT pups compared to control littermates. The diaphragm muscles of DMUT pups appeared shorter, and in most cases were readily detached from body wall (Fig. 2c–e, arrow).

During myogenesis, mononucleated myoblasts fuse to form multinucleated muscle cells that further differentiate to become mature myofibers. We examined the H&E-stained longitudinal sections of thigh and diaphragm muscles from postnatal day 1 (P1) DMUT mice and littermate controls. The control muscles were composed of elongated, cylindrical, well-aligned, and compact myofibers that were filled with striated myofibrils, whereas the mutant myofibers were markedly reduced in size and disconnected from each other (Fig. 2f–j). Many mutant muscle cells appeared spindle-shaped without a clear striated pattern (Fig. 2g). Although both control and DMUT myofibers were multinucleated, quantitative analysis revealed a significant reduction in the number of nuclei in PINCH mutant myofibers, suggesting impaired myoblast fusion (Fig. 2k). To examine whether decreased nuclear number in PINCH mutant myofibers could result from a change in the nuclear division, we performed immunostaining with a mitosis-specific antibody to phosphohistone H3 (Ph3) and observed instead a significant increase in mitotic nuclei in P1 DMUT muscle fibers compared to that in controls (Fig. 2l). In addition, significantly increased apoptosis was also observed in DMUT muscle cells (Fig. 2m).

MyoD is expressed early during myogenesis in myogenic progenitors and undifferentiated myoblasts and is required for muscle lineage commitment. To determine whether PINCHs play a role in early myogenesis, we traced skeletal muscle lineage (MyoD-Cre +) by β-galactosidase (β-gal) staining of E13.5 DMUT and control mice (on Rosa-lacZ background). We found that the overall shape and size of somites and muscle primordial blocks of the head, body, and limbs were comparable between PINCH mutant and control mice (Fig. 2n–q), suggesting that PINCH is dispensable for early stage myogenesis, but it is required for subsequent differentiation and maturation of muscle cells.

## Defects in myoblast fusion and cytoskeleton assembly in PINCH double mutant mice

**Fig. 3:**
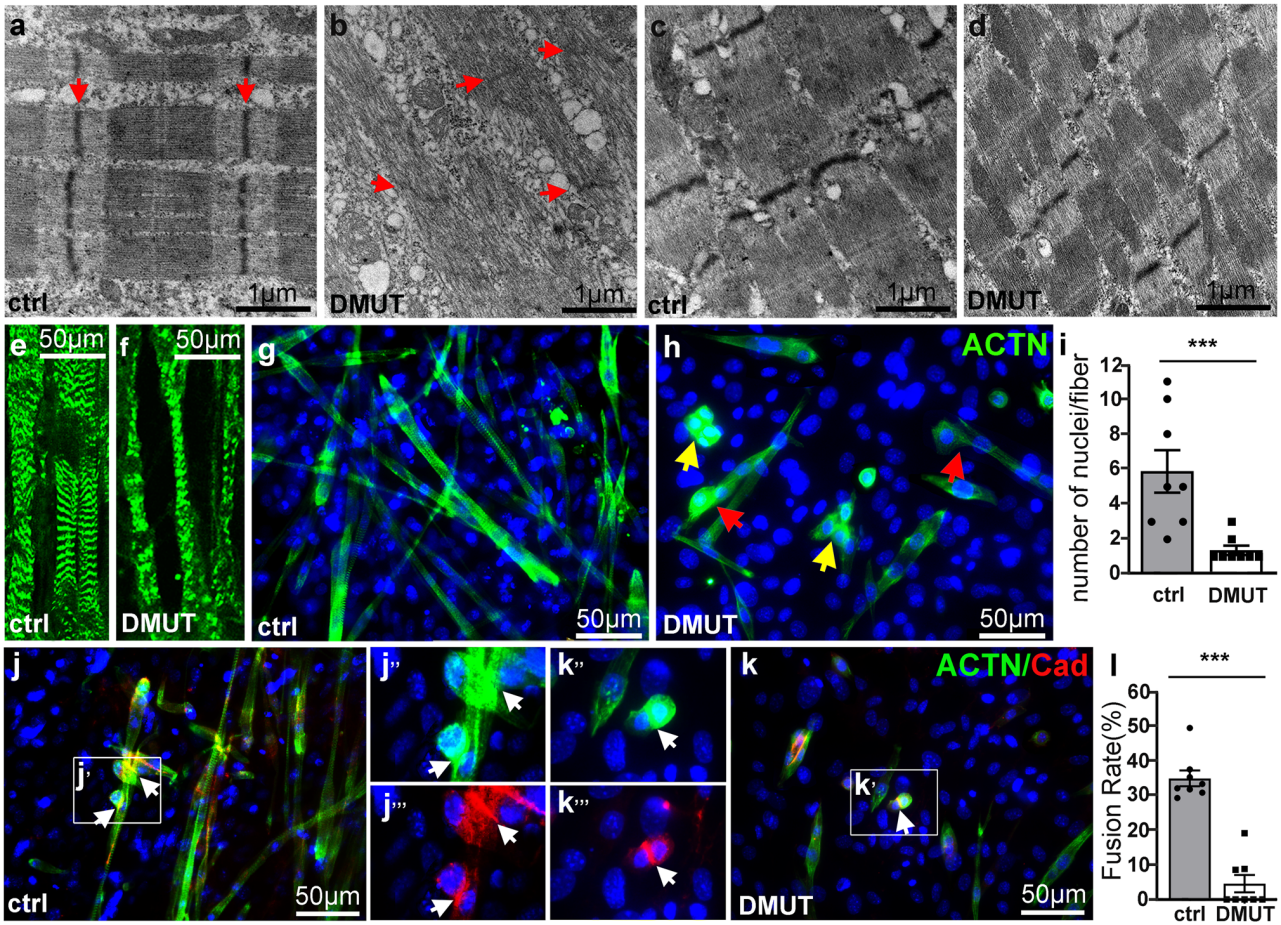
Defects in myoblast fusion and cytoskeleton assembly in PINCH double mutant mice. **a**–**d** Transmission electron microscopy (TEM) analysis of the limb **a**, **b **and diaphragm **c**, **d **of P1 DMUT and control muscles. Red arrow in A and B showing Z-line. **e**, **f** α-actinin immunostaining of hindlimb muscles showing defective assembly of myofibrils in DMUT mice at P1. **g**–**i** α-actinin immunostaining of myoblasts and myotubes from hindlimb muscles of DMUT and control mice at E18.5, showing that a majority of DMUT myoblasts remained mono-or binucleated, and were markedly smaller and spindle-like with no clear striations (yellow arrow indicating aggregated cells; red arrow indicating myoblasts aligned to newly formed myotubes). Quantification of the number of nuclei in DMUT and control myofibers **i**. (*n* = 8, error bars represent ± SEM, ***p < 0.001, 2-tailed *t*-test). **j**–**k** α-actinin and N-cad co-immunostaining showing expression of N-cadherin at the sites of cell fusion (arrow, boxed inlet **j**’, and **k**’). **l** Fusion rate, expressed as the percentage of nuclei included in myotubes (containing ≥ 3 nuclei), with respect to the total number of nuclei. The data were collected from at least eight independent experiments (error bars represent ± SEM, ***p < 0.001, 2-tailed *t*-test)

To further investigate the role of PINCH in sarcomere genesis, we examined the ultrastructures of muscle fibers by transmission electron microscopy (TEM). Control myofibers of the limb (Fig. 3a) and diaphragm (Fig. 3c) exhibited a typical striated pattern and well-organized sarcomere structures, including Z-and M-lines and I and A bands, and well-packed actin and myosin myofilaments (Fig. 3a–d). However, PINCH mutant limb myofibers largely lost their striated appearance with disorganized and truncated myofibrils. Sarcomere structures were severely disrupted, and only residual Z-lines and loosely packed myofilaments were visible (Fig. 3b, arrow). Sarcomere structure of PINCH mutant diaphragm myofibers appeared to be preserved; however, the myofibrils were markedly thinner and mal-aligned (Fig. 3d). Consistent with this, α-actinin immunostaining of hindlimb muscles showed that, compared to the controls, the myofibrils of the mutant myofibers were markedly thinner and lost their striated pattern (Fig. 3e, f), suggesting PINCH is essential for assembly of myofibril in vivo.

**Fig. 4:**
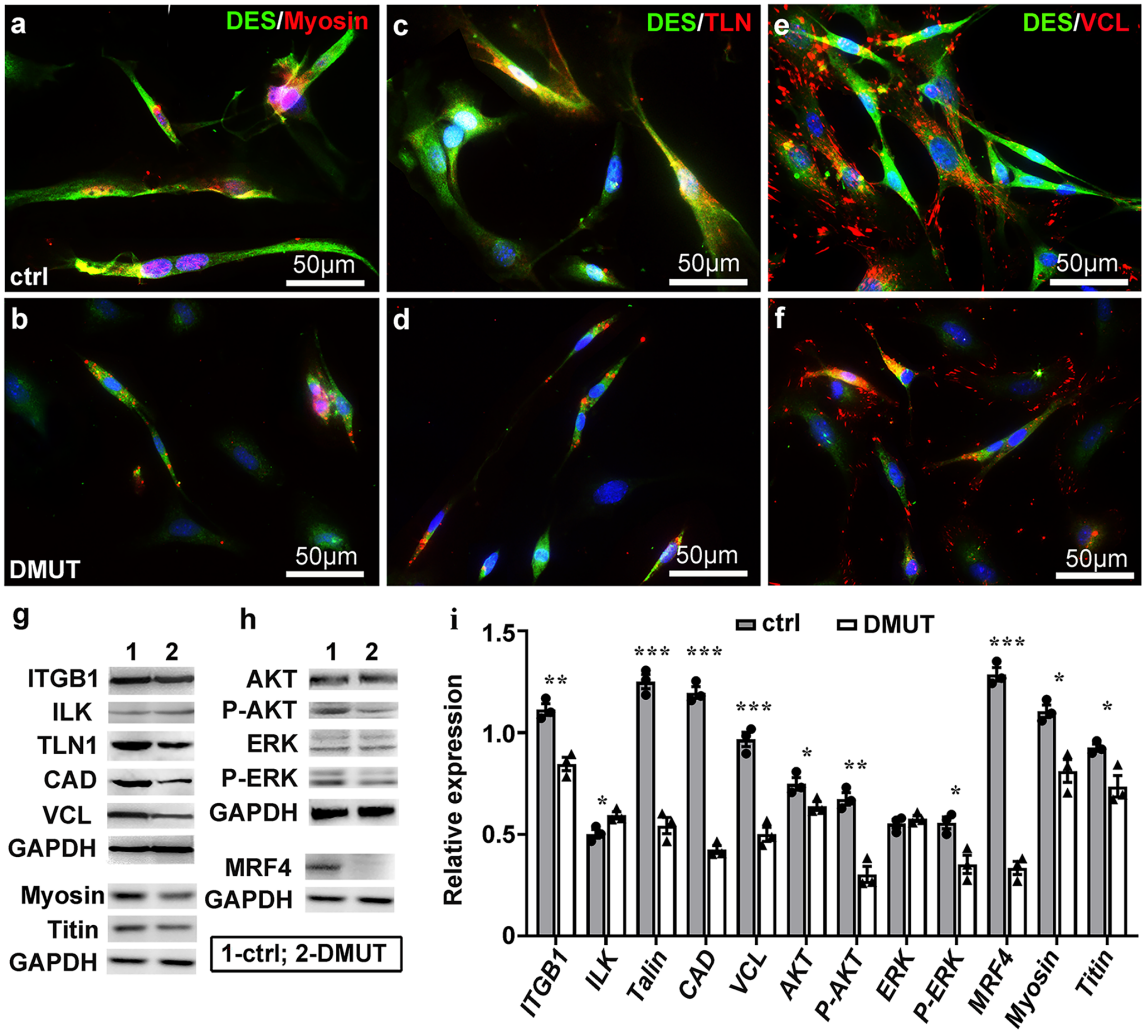
Defects in expression of cytoskeleton proteins and proteins involved in myogenesis in DMUT skeletal muscles. **a**–**f** DMUT and control myoblasts from E18.5 embryos were cultured for 48–72 h and co-immunostained to show expression of desmin (DES), Myosin, Talin (TLN) and Vinculin (VCL) during myoblast fusion and cytoskeleton assembly. **g**, **h** Western blot of protein lysates from DMUT and control quadriceps at P1. GAPDH was used as control. **i** The quantitative assessment of western blot bands (*n* = 3, *p < 0.05, **p < 0.01, ***p < 0.001, 2-tailed *t*-test, error bars represent ± SEM)

We further analyzed myoblast fusion and cytoskeleton assembly in vitro. Myoblasts were isolated from the hindlimb of control and DMUT mice at E18.5 and cultured in collagen-coated coverslips. After 3–5 days in culture, cells were fixed and co-immunostained with antibody to N-cadherin and αnd erins. We found that almost of the control myoblasts were fused, elongated, and become multinucleated myotubes with clear striated appearance (Fig. 3j). To quantify these observations, fusion rate was evaluated by counting the number of nuclei included in α-actinin-positive myotubes (containing ≥ 3 nuclei) divided by the total number of nuclei. The fusion rate in DMUT myoblasts cell cultures was reduced compared with control cells (Fig. 3l). However, a majority of DMUT myoblasts remained mono-or binucleated and were markedly smaller and spindle-like with no clear striations (Fig. 3h), even with extended culture time, which, however, resulted in excessive cell death in mutant cultures. Quantification revealed a significant reduction in the number of nuclei in mutant myofibers (Fig. 3i). However, we frequently observed cells that were aggregated (Fig. 3h yellow arrow) or aligned and fused to preexisting primary myofibers (Fig. 3h red arrow), suggesting those might be the cells in the process of cell fusion. In addition, expression of N-cadherins, known to regulate cell–cell adhesion and myoblast fusion, was correctly localized at the sites of cell contact and fusion, despite its level of expression was reduced (Fig. 3j, k, arrow, boxed inlet j’ and k’), suggesting PINCH may play a direct role in regulating myoblast fusion.

## Defects in expression of cytoskeleton proteins and proteins involved in myogenesis in DMUT skeletal muscles

Integrin signaling pathway regulates cytoskeleton organization and is required for skeletal myogenesis (Gheyara et al. 2007). Therefore, we analyzed the expression of proteins of integrin pathway and cytoskeleton and of those involved in late-stage myogenesis. DMUT and control myoblasts from E18.5 embryos were cultured for 48–72 h to avoid excessive cell death. Cells were fixed and immunostained with antibodies to desmin, myosin, talin, and vinculin to reveal the structures of cytoskeleton (Fig. 4a–f). DMUT muscle cells were markedly smaller and remained a myoblast-shape compared to controls (Fig. 4b, d, f), suggesting defects in cell spreading and cytoskeleton remodeling. In control muscle cells, expression of desmin, myosin, and talin were cytoskeleton-associated (Fig. 4a, c), and vinculin was concentrated at focal adhesion sites (Fig. 4e). In DMUT muscle cells, however, expression of myosin and talin were downregulated and aggregated (Fig. 4b, d). Vinculin was expressed in a pattern similar with that in control cells, although its expression was notably downregulated (Fig. 4f). These results suggested impaired assembly and organization of cytoskeleton but relatively normal adhesion of PINCH mutant cells. Consistent with this observation, western blot analysis of P1 mouse skeletal muscles revealed a significant decrease in the expression of several cytoskeleton-associated proteins, including β1-integrin, talin, N-cadherin, vinculin, myosin, and Titin (Fig. 4g–i). Interestingly, expression of ILK, known to interact with PINCH in focal adhesion complex, was slightly but significantly upregulated in DMUT, which might compensate partially for the loss of PINCH in cell adhesion. In DMUT skeletal muscles, phosphorylation of AKT and ERK1/2, the downstream mediators of integrin-focal adhesion, were significantly reduced, and total AKT is also slightly decreased. MRF4 is the predominant myogenic factor at the late stages of myogenesis and is required for myoblast fusion and cytoskeleton gene expression. Mutation of MYF4 is associated with centronuclear myopathy in humans. MRF4 expression was abolished in DMUT muscles that might contribute in part to observed defects in myoblast fusion and cytoskeleton organization.

## Discussion and conclusion

Skeletal muscle development is a multistep process involving complex cell–cell and cell-ECM interactions (Brown 1994; Clark et al. 2003; Mackinnon et al. 2002; Norman et al. 2007; Zervas et al. 2001). Progenitors that committed to skeletal myogenic fate delaminate and migrate to form the primordial muscle groups of the body. The majority of ECM molecules and their receptors (integrins and dystrophin) are expressed and play important roles in developing skeletal muscles. Despite their critical roles in cell adhesion and migration, initial muscle formation appears not to be disturbed in their absence. We found that the initial formation of muscle primordial masses in PINCH mutant mice was not disturbed. β1 integrin is the predominant beta subunit of integrin receptors, in its absence, however, migration and proliferation of myoblasts and initial muscle formation appear to be normal. Deletion of ILK leads to progressive myopathy at the adult stages. Similarly, β1 integrin, ILK, and PINCH are not required for the initial formation of myocardium. Instead, they are essential for the perinatal myocardial remodeling and maturation.

One of the unique features of skeletal myogenic differentiation is the fusion of mononucleated myoblasts to form multinucleated myotubes that further differentiate to become mature myofibers. Myoblast fusion is composed of several coordinated cellular processes including myoblast migration, adhesion, elongation, cell–cell recognition, alignment, and membrane fusion, and it is tightly controlled by the related genes and signaling pathways, including those involved in regulation of cytoskeleton dynamics, cell-ECM and cell–cell interactions. We found that, in PINCH mutant muscles, the size of myofibers and the number of myofiber nuclei were significantly decreased. Consistent with these in vivo observations, PINCH mutant myoblasts in culture were defective in spreading and elongation and appeared smaller. However, the mutant myoblasts were able to aggregate and align to primary myotubes but impeded at cell fusion. Expression of common cell adhesion molecules, such as N-cadherin and vinculin, was properly confined to the adhesion sites of PINCH mutant myoblasts, although the level of their expression was significantly reduced. These results have suggested a critical role of PINCHs in various aspects of myoblast fusion. Myoblast fusion defect in PINCH mutants closely resembles that observed in β1 integrin mutants. Similarly, β1 integrin deficient myoblasts can aggregate and express N-cadherin at cell adhesion sites correctly but stalled at cell membrane fusion. These results suggest that PINCH and β1 integrin regulate myoblast fusion by overlapping genetic pathways. In β1 integrin mutants, the expression of myogenic lineage markers is not changed, suggesting fusion defect in β1 integrin mutant myoblasts is not caused by defects in cell differentiation (Madaro et al. 2011). However, in PINCH mutants, expression of MRF4 is diminished, suggesting impaired myogenic differentiation may contribute to defective cell fusion in PINCH mutants. CD9 is known to be complex with β1 integrin and play a role in myoblast fusion and its expressed is abolished in β1 integrin mutants. In the future, it would be interesting to examine whether CD9 expression and its interaction with β1 integrin are disturbed and further investigate the potential crosstalk between CD9-and PINCH-mediated pathways in regulating myoblast fusion. Together, these results are consistent with the notion that multiple pathways are involved in myoblast fusion.

Our study has revealed a critical requirement for PINCH in regulating the assembly of the muscle fiber cytoskeleton. PINCH mutant myofibrils were markedly thinner and disorganized, and sarcomere structures were severely disrupted. Expression of cytoskeleton-associated proteins (β1-integrin, talin, N-cadherin, vinculin, myosin, and Titin) was significantly reduced. This defect is reminiscent of that observed in β1 integrin mutant muscles but distinct from that of skeletal muscle-specific ILK mutants, which exhibited an adult-onset muscular dystrophy. ILK interacts directly with the cytoplasmic domain of β1-integrins that facilitates the further recruitment and assembly of PINCH1-ILK-PARVIN (IPP) complex, which is required for protein stability and function of its component. Consistent with this, previous studies have shown that myocardial-specific ablation of PINCH1, β1-integrin, and ILK in mice causes similar dilated cardiomyopathy with disrupted sarcomere structures and aberrant expression of cytoskeleton-associated proteins. Ablation of PINCH in cardiomyocytes, as well as embryonic stem cells, results in reduced expression of ILK and vice versa. However, ablation of PINCH in neural crest cells does not affect ILK expression and vice versa (Dai et al. 2013; Liang et al. 2009b). PINCH1 expression is reduced in ILK mutant muscles, however, in our PINCH mutant muscles, expression of ILK is not reduced. Together, these data suggest that the interactome of ILK and PINCH and mutual dependence in their protein expression appear to be complex and context-dependent.

Muscle-specific ablation of PINCH1 caused only growth retardation and mild myopathy changes, likely due to the compensatory role by PINCH2 in skeletal muscles. PINCH1 mutants exhibited hypoplastic myofibers, split myofibers, and an increased number of centralized nuclei. Previous studies have shown that in many cases PINCH acts in the focal adhesion complex in the same genetic pathway as β1 integrin and ILK (Legate et al. 2006; Liang et al. 2009a, b, 2005; Wickström et al. 2010). In Drosophila and Caenorhabditis elegans, ablation of PINCH, ILK, and β1 integrin results in similar skeletal muscle phenotypes (Brown 1994; Clark et al. 2003; Lee et al. 2001; Mackinnon et al. 2002; Norman et al. 2007; Zervas et al. 2001). In contrast to that of β1 integrin and PINCHs, ablation of ILK in mouse skeletal muscle only resulted in progressive myopathy at adult stages, a phenotype similar with that observed in PINCH1 mutants, suggesting an additional mechanism may have evolved in skeletal myogenesis in mice that acts redundantly with ILK in binding integrins, which may warrant future study. In this context, it is relevant to note that talin and α-actinin, for instance, can bind to β1 integrins, thus linking integrins to the actin cytoskeleton in cell adhesions (Ellis et al. 2013).

Skeletal muscle development is critically regulated by a family of myogenic regulatory factors, among which, MRF4 is the most highly expressed MRF in muscles after birth (Weintraub et al. 1991; Zammit 2017). MRF4 plays an important role in myoblast fusion and maturation and muscle mass maintenance (Patapoutian et al. 1995). Genetic studies have revealed that ablation of MRF4c leads the myopathy with disorganized and significantly reduced myofibrils (Arnold and Winter 1998; Patapoutian et al. 1995). In human, mutation of MRF4 has been associated with myopathy and Becker muscular dystrophy (Kerst et al. 2001). Our study has suggested that PINCH is required for the expression of MRF4, which in turn promotes late-stage skeletal muscle differentiation and maturation.

In conclusion, our results demonstrated an essential role of PINCH in skeletal myogenic differentiation. Ablation of PINCH1 and PINCH2 in skeletal muscle progenitors resulted in impaired myoblast fusion and cytoskeleton organization. Expression of proteins involved in integrin pathway and cytoskeleton was significantly decreased in PINCH mutant skeletal muscles. Reduced expression of MRF4 might contribute to observed phenotypes in PINCH mutants.

## Data Availability

The data presented in this study are available in the current article.
